# Effects of Cebranopadol on Cocaine-induced Hyperactivity and Cocaine Pharmacokinetics in Rats

**DOI:** 10.1038/s41598-020-66250-z

**Published:** 2020-06-09

**Authors:** Huimei Wei, Linyue Shang, Chang-Guo Zhan, Fang Zheng

**Affiliations:** 1 Molecular Modeling and Biopharmaceutical Center, College of Pharmacy, University of Kentucky, 789 South Limestone Street, Lexington, KY 40536 USA; 2 Department of Pharmaceutical Sciences, College of Pharmacy, University of Kentucky, 789 South Limestone Street, Lexington, KY 40536 USA

**Keywords:** Molecular neuroscience, Molecular medicine

## Abstract

Cebranopadol is known as a highly potent analgesic. Recent studies also demonstrated that administration of cebranopadol significantly decreased cocaine self-administration and significantly reduced cue-induced cocaine-seeking behaviors in rats. However, it was unclear whether these interesting behavioral observations are related to any potential effects of cebranopadol on cocaine pharmacokinetics or cocaine-induced hyperactivity. In principle, a promising therapeutic candidate for cocaine dependence treatment may alter the cocaine pharmacokinetics and/or attenuate cocaine-induced reward and hyperactivity and, thus, decrease cocaine self-administration and reduce cue-induced cocaine-seeking behaviors. In this study, we examined possible effects of cebranopadol on cocaine pharmacokinetics and cocaine-induced hyperactivity for the first time. According to our animal data in rats, cebranopadol did not significantly alter the pharmacokinetics of cocaine. According to our more extensive locomotor activity testing data, cebranopadol itself also dose-dependently induced hyperactivity in rats at doses higher than 50 µg/kg. Cebranopadol at a low dose of 25 µg/kg (p.o.) did not induce significant hyperactivity itself, but significantly potentiated cocaine-induced hyperactivity on Days 4 to 7 after the repeated daily dosing of the drug.

## Introduction

Cebranopadol (CEB) is a novel, centrally acting analgesic which has been proven highly potent in various animal models of pain for preclinical studies and efficacious in multiple clinical trials^1^. To date, there have been 12 clinical trials including 11 completed trials (https://clinicaltrials.gov/ct2/results/details?term=cebranopadol). Cebranopadol, with a unique mode of action, is a high-affinity agonist at both μ-opioid peptide (MOP) receptor (*K*_d_ = 0.7 nM) and nociceptin opioid peptide (NOP) receptor (*K*_d_ = 0.9 nM), and can also activate κ-opioid peptide (KOP) receptor (*K*_d_ = 2.6 nM) and δ-opioid peptide (DOP) receptor (*K*_d_ = 18 nM) at higher concentrations^2^. Compared to classic opioid analgesics such as morphine and hydromorphone, cebranopadol is more effective and safer with a better tolerability and has less addiction potential in humans when used as an analgesic^3–6^.

Previous studies suggest that NOP receptor may be a potentially interesting target for treatment of drug dependence by attenuating the rewarding effects of cocaine and other drugs of abuse^7–9^. In addition, a growing body of evidence suggests that simultaneous targeting of multiple opioid receptors is necessary to reduce classic opioid side effects^10–12^. For this reason, with the characteristics of high activity at both the MOP and NOP receptors, along with multiple activation on other opioid receptors, cebranopadol has attracted much attention as a potential therapeutic candidate for treatment of cocaine dependence. Specifically, two recent studies demonstrated that administration of cebranopadol significantly decreased cocaine self-administration^13^ and significantly reduced cue-induced cocaine-seeking behaviors^14^ in rats. However, it was unclear whether these interesting behavioral observations are related to any potential effects of cebranopadol on cocaine pharmacokinetics or cocaine-induced hyperactivity. As noted in one of the reports^14^, these reported studies did not examine potential effects of cebranopadol on the pharmacokinetics of cocaine or its effects on cocaine-induced hyperactivity. In principle, a promising therapeutic candidate for cocaine dependence treatment may alter the pharmacokinetics (PK) of cocaine and/or attenuate cocaine-induced reward and hyperactivity (locomotor-stimulating effect)^15,16^ and, thus, decrease cocaine self-administration and reduce cue-induced cocaine-seeking behaviors.

As well-known, locomotor activity test is a basic unconditioned behavioral model to study the psychoactive effects of testing compounds^17^. Psychostimulants and opioids directly or indirectly promote neuronal activity in mesocorticolimbic dopamine (DA) system to increase extracellular DA in the brain^18,19^. Hence, dopaminergic systems are crucially involved in locomotor-stimulating effects and physical dependence in addiction of psychostimulants and the opiates^20–23^. Despite of the considerable studies on the analgesic effects of cebranopadol^1,2,24,25^, the actions of cebranopadol on spontaneous motor activity of rats have not been characterized extensively. De Guglielmo *et al*.^14^ did a locomotor activity test in rats administered orally of 25 µg/kg cebranopadol, and concluded that cebranopadol did not affect the locomotor activity. To the best of our knowledge, there has been no report of studies on the impact of cebranopadol on cocaine-induced hyperactivity. It is also interesting to know whether cebranopadol alters the PK profile of cocaine, as animal behavioral data are closely correlated with the PK of psychostimulants^26–28^.

In this study, we examined the possible effects of cebranopadol on cocaine pharmacokinetics and cocaine-induced hyperactivity (locomotor-stimulating effect). According to our animal data in rats, cebranopadol did not significantly alter the PK of cocaine. Further, cebranopadol significantly potentiated cocaine-induced hyperactivity at least after repeated drug administration, which is unexpected as the previous study^14^ revealed that cebranopadol did not affect the locomotor activity in rats. In fact, according to our more extensive locomotor activity testing data, cebranopadol itself also dose-dependently induced hyperactivity in rats at doses higher than 50 µg/kg.

## Results

### Influence of procedure manipulation on locomotor behavior

**Figure 1:**
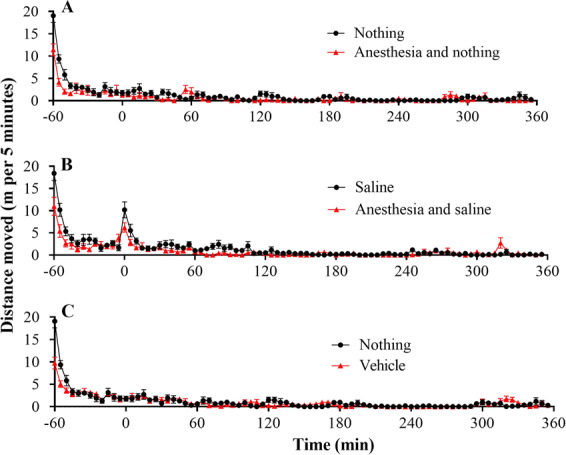
Baseline locomotor activity of rats (n = 12) under various testing environments/procedures. (**A**) Effects of anesthesia without administration of saline or vehicle. (**B**) Effects of anesthesia with saline administration; saline (1 ml/kg) was injected (i.p.) at *t* = 0 (*i.e*. 60 minutes after the rats were put into the chambers). (**C**) Effects of vehicle (corn oil) without anesthesia; vehicle oil (1 ml/kg) was administered (p.o.) 15 min before the rats were put into the testing chambers. “Nothing” means no administration of saline or vehicle. Data are plotted as mean ± SEM meters moved per five minutes. The data were analyzed by two-way ANOVA with *post hoc* analysis, showing no significant difference between any of the pairs.

**Figure 2:**
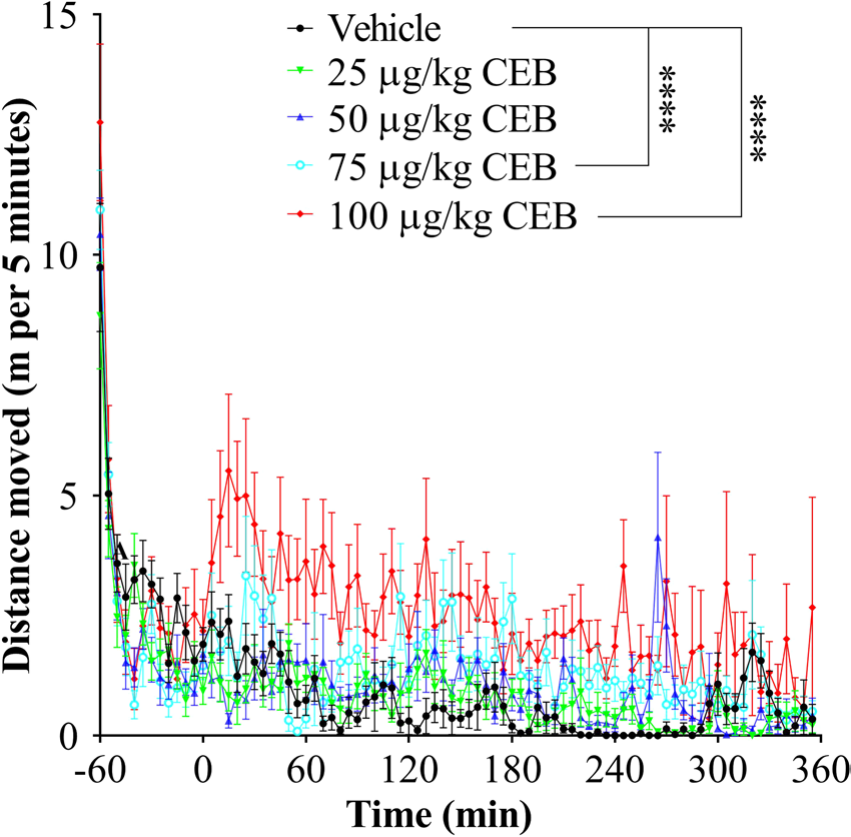
Hyperactivity in the same group of rats (n = 12) induced by cebranopadol (CEB) at various doses including 0 (vehicle), 25, 50, 75, and 100 µg/kg CEB in ascending order. Each time, vehicle or CEB was administered (p.o.) 15 min before the rats were put into the testing chambers. Data are plotted as mean ± SEM meters moved per five minutes and statistical significances were analyzed by two-way ANOVA with *post hoc* analysis.

**Figure 3:**
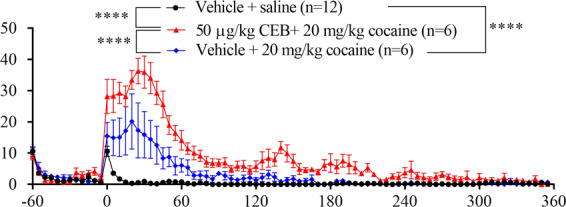
Effects of 50 µg/kg cebranopadol (CEB) on cocaine-induced hyperactivity. The 12 rats were assigned randomly to two groups: testing and control groups (n = 6 per group). CEB (for the testing group) or vehicle (oil for the control group) was administered (p.o.) 15 min before the rats were put into the testing chambers; cocaine (20 mg/kg, i.p.) were administered at *t* = 0 (*i.e*. 60 minutes after the rats were put into the chambers). Prior to the locomotor activity testing with cocaine, the baseline locomotor activity data with vehicle + saline (1 ml/kg, i.p.) were determined in both groups of the rats (n = 6 + 6 = 12). Data are plotted as mean ± SEM meters moved per five minutes and statistical significances were analyzed by two-way ANOVA with *post hoc* analysis.

**Figure 4:**
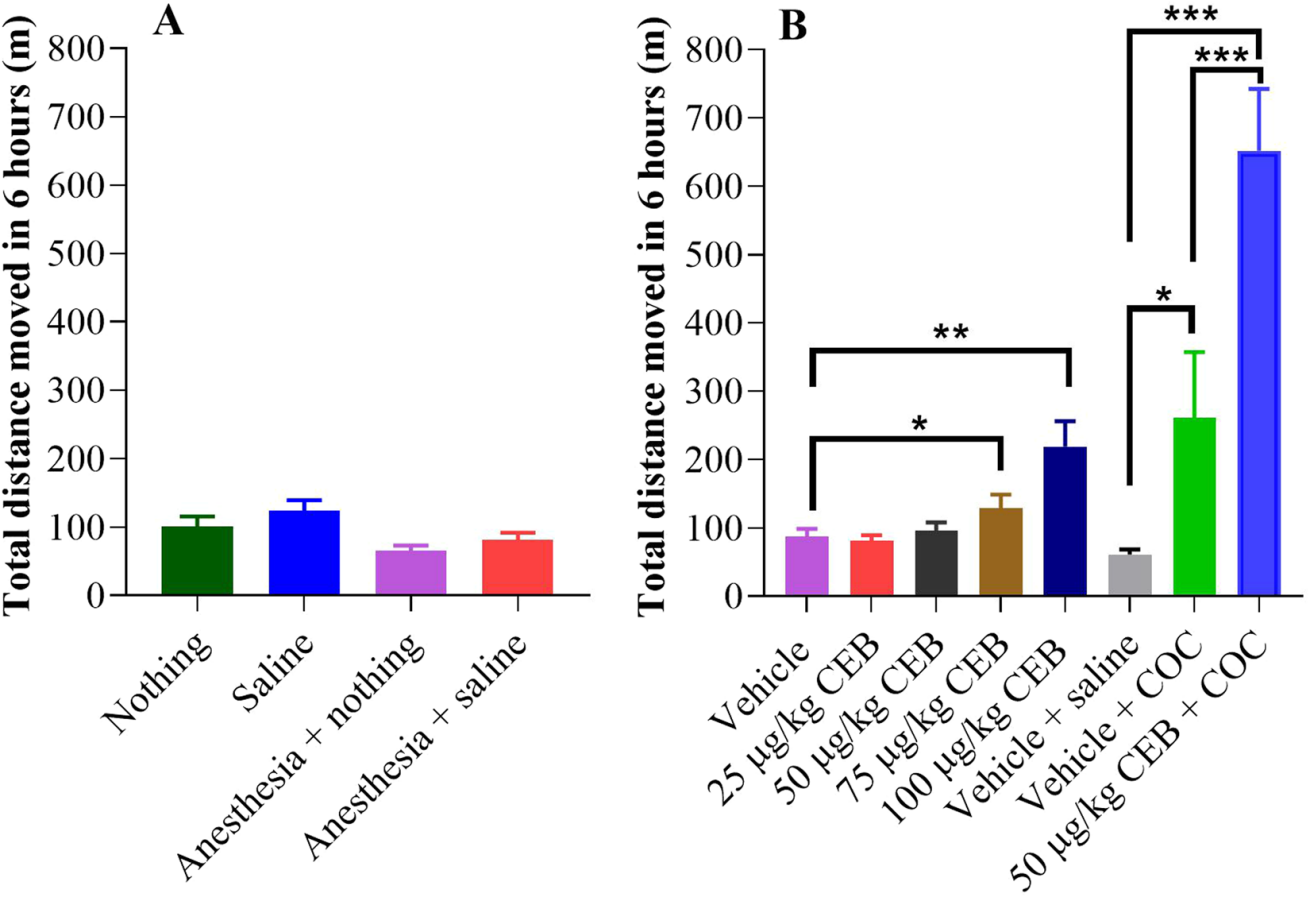
Total distances moved in 6 hours after cebranopadol (CEB) and/or cocaine (COC at a dose of 20 mg/kg, i.p.) administration in rats (n = 12). (**A**) Various negative control conditions; (**B**) dose-dependent effects of CEB on locomotor activity and effects of CEB on cocaine-induced hyperactivity. Data are presented as mean ± SEM and statistical significances were analyzed by one-way ANOVA with *post hoc* analysis.

**Figure 5:**
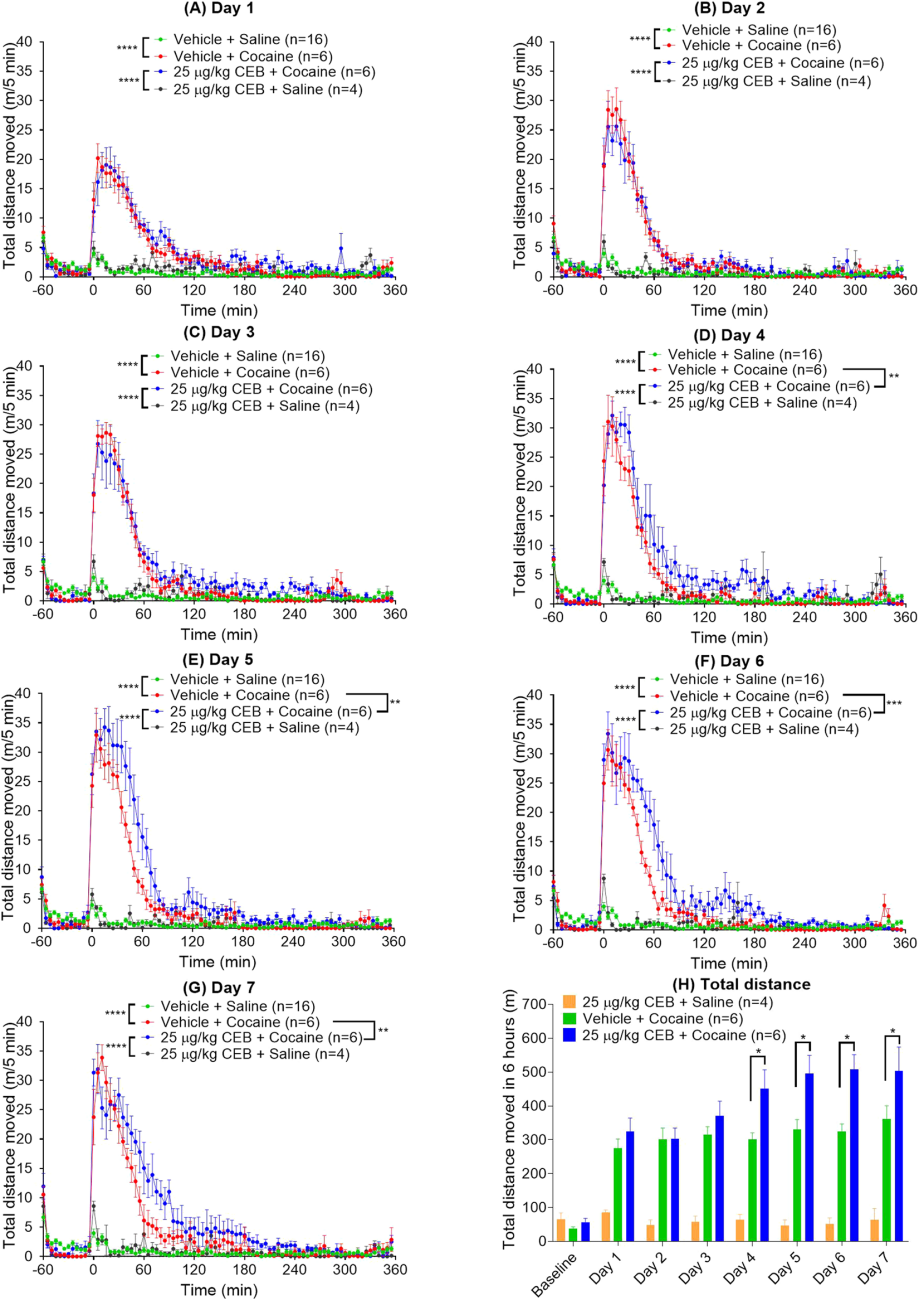
Effects of 25 µg/kg cebranopadol (CEB) on cocaine-induced hyperactivity. These additional locomotor activity tests were performed on other 16 rats with daily administration of cocaine (20 mg/kg, i.p.) alone (n = 6) or cebranopadol (25 µg/kg, p.o.) + cocaine (20 mg/kg, i.p.) (n = 6) or cebranopadol (25 µg/kg, p.o.) (n = 4) alone within a week: (**A**) Day 1; (**B**) Day 2; (**C**) Day 3; (**D**) Day 4; (**E**) Day 5; (**F**) Day 6; (**G**) Day 7. Panel (**H**) shows the total distance traveled in six hours after the cocaine (or saline) administration. Prior to any drug (cocaine and/or cebranopadol) administration, all rats (n = 6 + 6 + 4 = 16) were tested for the baseline locomotor activity (with saline instead of cocaine) on Day 0 (baseline). The same baseline locomotor activity data (n = 16) were indicated in panels A to G as the common negative control. Each day, CEB or vehicle (oil) was administered (p.o.) 15 min before the rats were put into the testing chambers; cocaine (20 mg/kg, i.p.) was administered at *t* = 0 (*i.e*. 60 minutes after the rats were put into the chambers). Data are plotted as mean ± SEM meters moved per five minutes and statistical significances were analyzed by two-way ANOVA with *post hoc* analysis.

We carried out extensive locomotor activity tests (see Figs. 1–5) to examine whether cebranopadol has any effects on the locomotor activity of rats with or without cocaine administration. First of all, we examined possible effects of injection procedure, anesthesia, and vehicle (corn oil) on the locomotor activity in 12 rats during the first several days in order to habituate rats to the injection procedure and exclude manipulation influence. As shown in Fig. 1, curves plotted with distance moved per five minutes after anesthesia and vehicle treatment were overlapped with the corresponding control groups, showing no significant differences between the groups in the total distance moved in six hours from time 0 to 360 min (see Figs. 1 and 4A), as expected.

### Cebranopadol-induced locomotor activity

Rats, administered with an ascending dose (25, 50, 75, or 100 µg/kg, p.o.) of cebranopadol, were placed in non-porous plastic chambers to observe toxicity signs and monitor locomotor activity induced by cebranopadol. It turned out that cebranopadol did not elicit locomotor activity by itself when the dose was low (25 or 50 µg/kg; see Fig. 2). However, as the dose increased, the animals became apparently more active (Fig. 2). According to the two-way analysis of variance (ANOVA) with *post hoc* analysis, both 75 and 100 µg/kg cebranopadol induced significant hyperactivity (*p* < 0.0001). These observations suggest that the rats are more active after treatment with 75 or 100 µg/kg cebranopadol than with 25 or 50 µg/kg cebranopadol during the testing process. Consistently, as shown in Fig. 4B, one-way ANOVA with *post hoc* analysis (on the total distances moved within six hours) also revealed a significant increase in total distance moved in six hours following the treatment with 75 and 100 µg/kg cebranopadol (*p* < 0.05 and *p* < 0.01, respectively) compared to the vehicle treatment and 50 µg/kg cebranopadol. We did not observe any adverse effects during the entire testing process.

### Cebranopadol potentiated cocaine-induced hyperactivity

The dose of 50 µg/kg cebranopadol (p.o.) was chosen to investigate the effect of cebranopadol on cocaine-induced hyperactivity because it was the highest dose without inducing hyperactivity on its own, as noted above. 20 mg/kg cocaine (i.p.)-treated rats showed distinct hyperactivity (Fig. 3, *p* < 0.0001 *vs* the vehicle baseline), and they calmed down in about two hours after the cocaine injection. Two-way ANOVA with *post hoc* analysis revealed significantly increased locomotor activity after co-administration of 20 mg/kg cocaine (i.p.) and 50 µg/kg cebranopadol (p.o.) (Fig. 3, *p* < 0.0001) in comparison with the injection of 20 mg/kg cocaine (i.p.) alone. The locomotor-stimulating effect sustained for a longer period of time during which the rats were not even calmed down until four hours after the cocaine administration. One-way ANOVA with *post hoc* analysis indicated that pretreatment with 50 µg/kg cebranopadol (p.o.) augmented cocaine-induced hyperactivity significantly, more than two times higher compared to the treatment with cocaine alone and more than six times higher compared to the treatment with 50 µg/kg cebranopadol by analyzing total distance moved in six hours (Fig. 4B).

It should be noted that the above-discussed effects of 50 µg/kg cebranopadol on cocaine-induced hyperactivity were based on the locomotor activity testing in the same 12 rats that had been tested with cebranopadol (as reported in Table 1). A potential question was whether the previous cebranopadol administration would significantly affect the subsequent locomotor activity with cebranopadol again. Further, because previous studies^13,14^ demonstrated that cebranopadol at the dose of 25 µg/kg (p.o.) significantly decreased cocaine self-administration, would cebranopadol at a dose of 25 µg/kg (p.o.) also potentiate cocaine-induced hyperactivity? To address these questions, we also carried out further locomotor activity tests on other 16 rats with daily administration of cocaine (20 mg/kg, i.p.) alone or cebranopadol (25 µg/kg, p.o.) + cocaine (20 mg/kg, i.p.) or cebranopadol (25 µg/kg, p.o.) alone during a week.

**Table 1:** Locomotor activity testing schedule (n = 12 rats).

Day of Study	Manipulation	Number of Rats/Group in Testing	Experimental Purpose
1	Acclimation for 3 hours	n = 12	Habituate the rats to injection procedures and exclude manipulation influence
3	7-hour test without manipulation	n = 12	
5	Saline	n = 12	
7	Anesthesia	n = 12	
9	Anesthesia + saline	n = 12	
11	Vehicle	n = 12	Investigate cebranopadol-induced locomotor activity
13	25 µg/kg Cebranopadol	n = 12	
15	50 µg/kg Cebranopadol	n = 12	
17	75 µg/kg Cebranopadol	n = 12	
19	100 µg/kg Cebranopadol	n = 12	
21	Vehicle + saline	n = 12	Examine impact of cebranopadol on cocaine-induced hyperactivity
23	Vehicle + cocaine	Randomly selecting 6 rats	
24	50 µg/kg Cebranopadol + cocaine	The other 6 rats left	

As seen in Fig. 5, repeated daily dosing of 25 µg/kg cebranopadol (p.o.) within a week never induced significant hyperactivity; there was no significant difference in the locomotor activity among all the locomotor activity tests with 25 µg/kg cebranopadol or saline (baseline), according to two-way ANOVA with *post hoc* analysis. Further, based on the results depicted in Fig. 5A to **C**, 25 µg/kg cebranopadol (p.o.) did not significantly change cocaine-induced hyperactivity during the locomotor activity tests after the drug (cocaine and/or cebranopadol) administration on Days 1 to 3. There was no significant difference in the hyperactivity between the two groups (cocaine alone group and cocaine + cebranopadol group) on Days 1 to 3. However, during Days 4 to 7, compared to the hyperactivity induced by administration of cocaine (20 mg/kg, i.p.) alone, the hyperactivity induced by co-administration of cocaine (20 mg/kg, i.p.) and cebranopadol (25 µg/kg, p.o.) had a nearly equal (or even slightly lower) peak, but lasted a longer period of time (see Fig. 5D–G). The difference was significant on Days 4 to 7, based on two-way ANOVA with *post hoc* analysis. We also performed one-way ANOVA with *post hoc* analysis on the total distance moved during six hours after the drug administration, showing that the total distance associated with the co-administration of cocaine (20 mg/kg, i.p.) and cebranopadol (25 µg/kg, p.o.) was significantly longer than that associated with administration of cocaine (20 mg/kg, i.p.) alone on Days 4 to 7 (Fig. 5H).

### Cebranopadol did not change the PK profile of cocaine

**Figure 6:**
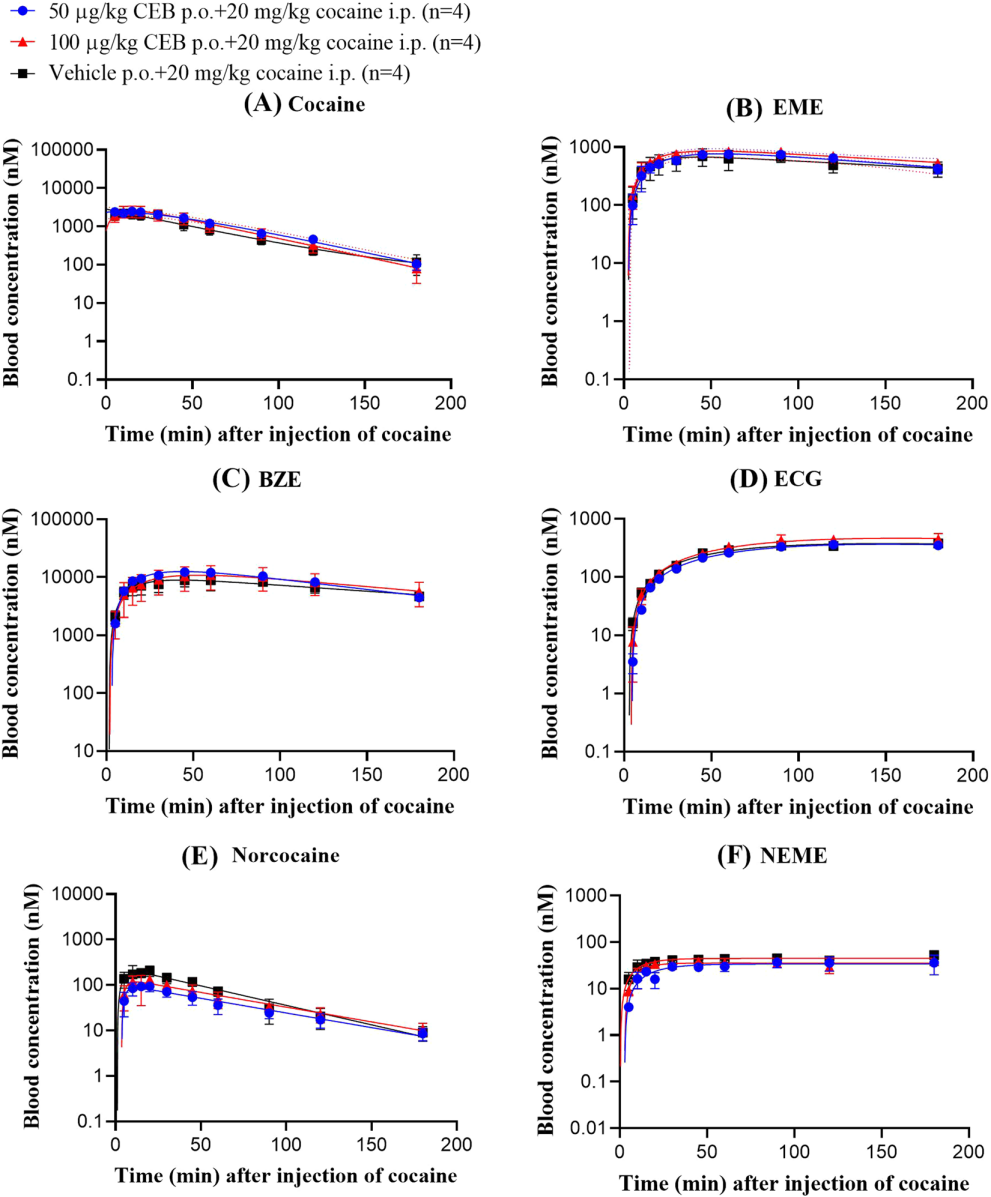
Impact of cebranopadol on the PK profile of cocaine in rats (n = 4 per group). Time-dependent concentrations of (**A**) Cocaine, (**B**) EME, (**C**) BZE, (**D**) ECG, (**E**) Norcocaine, and (**F**) NEME. Rats received nothing (for control group), 50 or 100 µg/kg cebranopadol (p.o. for testing groups) 60 min before cocaine administration (20 mg/kg, i.p.). The blood concentrations of cocaine and its metabolites were detected simultaneously. BZE, EME, NEME, and ECG are abbreviation of benzoylecgonine, ecgonine methyl ester, norecgonine methyl ester, and ecgonine, respectively. Data are plotted as mean ± SEM and statistical significances were analyzed by two-way ANOVA with *post hoc* analysis.

To investigate whether cebranopadol can significantly alter the pharmacokinetic profile of cocaine and time course in plasma or attenuate/potentiate cocaine-induced hyperactivity, we conducted PK study to measure the concentrations of cocaine and all its metabolites in whole blood after pretreatment of cebranopadol. As showed in Fig. 6, we did not detect any significant effects of cebranopadol on cocaine PK profile according to our two-way ANOVA for the time courses of the blood concentrations of each compound (cocaine or its metabolite). Therefore, the increase of cocaine-induced hyperactivity associated with the pretreatment of cebranopadol was not caused by changing the blood concentrations of cocaine and its active metabolites.

## Discussion

According to the locomotor activity data, cebranopadol at a low oral dose of 25 or 50 µg/kg did not significantly affect the locomotor activity in rats. The previously reported study also observed that cebranopadol at an oral dose of 25 µg/kg did not affect general locomotor activity of the rats^14^. However, 25 µg/kg cebranopadol (p.o.) was the only dose tested in the previous study^14^. The present study revealed that cebranopadol dose-dependently stimulates locomotor activity in rats, demonstrating significant hyperactivity at an oral dose of 75 or 100 µg/kg, but without showing any visible toxicity signs during the locomotor activity tests. The observed dose-dependent hyperactivity induced by cebranopadol is actually consistent with the previously reported observation that cebranopadol had discriminative stimulus property^29^ to certain extent like morphine and the fact that cebranopadol is also an MOP receptor agonist (*K*_d_ = 0.7 nM) which is expected to induce hyperactivity at a sufficiently high dose^30,31^.

It is interesting to note that, according to our locomotor activity data, 25 µg/kg cebranopadol (p.o.) did not induce significant hyperactivity itself, but significantly potentiated cocaine-induced hyperactivity on Days 4 to 7 after the repeated daily dosing the drug. As well know, cocaine induces a uniformly dose-dependent hyperactivity after acute administration^32,33^. Concerning why an opioid can potentiate cocaine-induced hyperactivity, a number of preclinical studies have revealed cross-sensitization between opioids and cocaine, demonstrating that some other opioids also clearly potentiate cocaine-induced hyperactivity^34–37^. In general, both a psychostimulant, such as cocaine, and an opioid activate or augment the dopaminergic input into the nucleus accumbens and frontal cortex to produce psychomotor stimulant actions^34–36^. In particular, Smith *et al*.^37^ examined cross-sensitization between opioids and cocaine, and determined that the extent of cross-sensitization was mediated by an opioid’s selectivity for MOP, KOP, and DOP receptors. Likewise, their results revealed that MOP agonists morphine and buprenorphine and DOP agonist BW373U86 produced synergistic effects in cocaine locomotor activity tests. Cebranopadol may follow the same rule as it is a potent agonist of both NOP receptor (*K*_d_ = 0.9 nM) and MOP receptor (*K*_d_ = 0.7 nM).

Concerning why cebranopadol is capable of significantly decreasing cocaine self-administration and reducing cue-induced cocaine-seeking behaviors while significantly potentiating cocaine-induced hyperactivity, one possible explanation is that cebranopadol and buprenorphine have the similar effects. In fact, buprenorphine attenuates the expression of cocaine sensitization and other cocaine-related behaviors by increasing basal levels of glutamate in the nucleus accumbens, which serves to decrease the effectiveness of cocaine or cocaine-associated cues^38^.

We also investigated whether the alteration of pharmacokinetics (PK) of cocaine after cebranopadol administration was the possible reason for cebranopadol potentiating cocaine-induced hyperactivity. The metabolic pathways of cocaine have been well known^15,39–41^. Briefly, majority of cocaine (>90%) in rodents and primates is hydrolyzed to benzoylecgonine (BZE) and biologically inactive metabolite ecgonine methyl ester (EME) by either carboxylesterases^42^ or butyrylcholinesterase (BChE)^16,43,44^, and only minor portion (~5%) is oxidized into norcocaine (NC) by P450^45–47^. Concerning physiological effects of BZE, early investigation indicated that it was a potent vasoconstrictor, whereas more recent literature suggested that it was an inactive metabolite of cocaine^48,49^. NC is more toxic than cocaine itself, causing the hemodynamic changes and producing lethality^49–51^. Plasma BChE can further hydrolyze BZE to ecgonine (ECG) and NC to norecgonine methyl ester (NEME)^45,47,52,53^. Among all these compounds, only cocaine but not NC produced stimulatory locomotor activity^51^, whereas EME and ECG resulted in no specific effect^26^. According to our PK data, cebranopadol did not affect the peripheral processing or general accessibility of cocaine to the brain to activate motor systems after co-administration of cebranopadol and cocaine, which indicates that the behavioral differences observed after co-administration of cebranopadol and cocaine cannot be attributed to the change in the cocaine PK profile. In fact, cocaine concentration determined in blood is well correlated with the presence of cocaine concentration in the brain^54^. In mice, the brain/blood concentration ratios of cocaine and BZE, on the average, were approximately 7 and 0.1, respectively, between 5 and 60 min after i.p. administration of cocaine^54^; in human fatal cases, the brain/blood concentration ratios were 9.6 for cocaine and 0.36 for BZE^55^.

Further, compared to duration of the hyperactivity induced by cocaine alone, the duration of time for the hyperactivity induced by co-administration of cebranopadol and cocaine was much longer. The prolonged duration was likely related to the longer duration of action of cebranopadol. Notably, there has been no study reporting the concentration of cebranopadol in blood or brain (PK profile) and duration of action after oral administration of cebranopadol in rats. In fact, except for some professional pharmaceutical companies, it is difficult for a lab to determine the PK profile of cebranopadol administered orally, because its effective oral dose is too low to produce a concentration in plasma for a commonly used instrument to detect the compound. Nevertheless, a previous study revealed that the duration of action after intravenous (i.v.) administration of 12 µg/kg cebranopadol lasted for up to 7 hours (much longer than morphine at the equivalent effective dose) and the half-life of cebranopadol with a high i.v. dose of 160 µg/kg was 4.52 hours in rats^2^.

In summary, the findings in the present study suggest that cebranopadol can dose-dependently induce weak hyperactivity on its own, and that cebranopadol at a low dose of cebranopadol (25 µg/kg, p.o.) which does not induce significant hyperactivity by itself can significantly potentiate cocaine-induced hyperactivity at least after the repeated daily dosing of the drug. These findings should arouse caution that cebranopadol could have some dose-dependent rewarding/positive reinforcement effects. In consideration of the important role of dopaminergic systems involved in locomotor-stimulating effects and physical dependence in addiction of psychostimulants and the opioids, more extensive studies are needed to understand the effects of cebranopadol on dopaminergic systems. A clear understanding of the potential of rewarding and positive reinforcement effects of this compound underlying the psychological and physical behavior is required before further clinical development of the compound in treatment of cocaine-dependent patients. This knowledge will also be valuable for using cebranopadol in clinical trials as an analgesic.

## Materials and methods

### Animals and drugs

Male Sprague-Dawley rats were ordered from Harlan (Harlan, Indianapolis, IN), and housed initially in two rats per cage. Unless explicitly indicated otherwise, all rats were allowed *ad libitum* access to food and water except during testing time and maintained on a 12 h light/12 h dark cycle, with the lights on at 7:00 am at constant room temperature (21–22 °C) and humidity (45–55%)^56,57^. All experiments were performed during the light phase of the light/dark cycle in accordance with the Guide for the Care and Use of Laboratory Animals as adopted and promulgated by the National Institutes of Health. The animal protocol was approved by the IACUC (Institutional Animal Care and Use Committee) at the University of Kentucky. Cebranopadol (Sigma Aldrich, USA) was freshly prepared as a fine suspension with 100% corn oil by ultrasonic and administered by gavage (p.o.). (-)-Cocaine hydrochloride was provided by the National Institute on Drug Abuse (NIDA) Drug Supply Program (Bethesda, MD) and was dissolved in sterile saline with intraperitoneal (i.p.) administration. Paraoxon and heparin were purchased from Thermo Fisher Scientific (Waltham, MA). Formic acid was from Sigma-Aldrich (St. Louis, MO).

### Locomotor activity testing procedure

Cebranopadol- and cocaine-induced locomotion activity was monitored individually by using a video-tracking system (as described previously^56,58^) with rats weighing 260–320 grams at the beginning. Briefly, the locomotor activity tests were performed in high-density, non-porous plastic chambers measuring 50 cm (L) × 50 cm (W) × 38 cm (H) in a light- and sound-attenuating behavioral test enclosure (San Diego Instruments, San Diego, CA). Cumulative distance traveled was recorded by ANY-maze video tracking system (San Diego Instruments, San Diego, CA) to assess the locomotor activity beginning at 9:00 on testing day. The distance traveled was collected in 5-min bins. Cebranopadol or vehicle (oil) was p.o. administered 15 min under minor anesthesia with isoflurane before the rats were put into the testing chambers for 7-hour (*i.e*. time −60 to 360 min interval at X-axis) video track with or without administration of sterile saline (1 ml/kg, i.p.) or cocaine (20 mg/kg, i.p.) at 60 min (*i.e*. time 0 min point at the X-axis) after the rats were put into the testing chambers. Locomotor activity tests on the first 12 rats were proceeded as timeline in Table 1 and all injections were performed very gently. On non-testing days, all rats stayed in their home cages located in the rat-housing room. In addition to the locomotor activity tests listed in Table 1, further locomotor activity tests were performed on other 16 rats with daily administration of cocaine (20 mg/kg, i.p.) alone (n = 6) or cebranopadol (25 µg/kg, p.o.) + cocaine (20 mg/kg, i.p.) (n = 6) or cebranopadol (25 µg/kg, p.o.) (n = 4) alone within a week.

### Metabolism of cocaine after treatment with cebranopadol

Rats received a dose of 1 ml/kg vehicle, 50 or 100 µg/kg cebranopadol at 60 min before cocaine administration (20 mg/kg, i.p.). Blood samples (75 µl/sample) were collected from saphenous veins into heparin-treated capillary tubes at 5, 10, 15, 20, 30, 45, 60, 90, 120, and 180 min after the cocaine injection, and mixed with 100 µl paraoxon solution (250 µM paraoxon with 10 U/ml heparin in 0.1% formic acid) immediately. Blood samples were stored at −80 °C until analysis by using our previously developed LC-MS/MS method^52^ for simultaneously detecting the concentrations of cocaine and its metabolites in blood samples.

### Statistical analysis

All data were analyzed statistically with the Prism 8 software (GraphPad Software, San Diego, CA). All data are presented as mean ± SEM. One-way analysis of variance (ANOVA) with *post hoc* analysis was used to analyze differences of total distance moved in six hours in locomotion study. Two-way ANOVA with *post hoc* analysis was used to analyze the distance moved per 5 minutes in locomotor activity testing and the effects of cebranopadol on cocaine PK profile. Statistical significance was set at *(*p* < 0.05), **(*p* < 0.01), ***(*p* < 0.001), or ****(*p* < 0.0001).

## References

[1] Tzschentke TM, Linz K, Koch T, Christoph T (2019). Cebranopadol: A Novel First-in-Class Potent Analgesic Acting via NOP and Opioid Receptors. Handb. Exp. Pharmacol..

[2] Linz K (2014). Cebranopadol: a novel potent analgesic nociceptin/orphanin FQ peptide and opioid receptor agonist. The Journal of pharmacology and experimental therapeutics.

[3] Gohler K (2019). Assessment of the Abuse Potential of Cebranopadol in Nondependent Recreational Opioid Users: A Phase 1 Randomized Controlled Study. J Clin Psychopharmacol.

[4] Eerdekens MH (2019). Cancer-related chronic pain: Investigation of the novel analgesic drug candidate cebranopadol in a randomized, double-blind, noninferiority trial. European journal of pain (London, England).

[5] Tzschentke TM, Kogel BY, Frosch S, Linz K (2018). Limited potential of cebranopadol to produce opioid-type physical dependence in rodents. Addict. Biol..

[6] Ruzza C, Holanda VA, Gavioli EC, Trapella C, Calo G (2019). NOP agonist action of cebranopadol counteracts its liability to promote physical dependence. Peptides.

[7] Zhao RJ (2003). Orphanin FQ/nociceptin blocks methamphetamine place preference in rats. Neuroreport.

[8] Sakoori K, Murphy NP (2008). Endogenous nociceptin (orphanin FQ) suppresses basal hedonic state and acute reward responses to methamphetamine and ethanol, but facilitates chronic responses. Neuropsychopharmacology.

[9] Ciccocioppo R (2004). Attenuation of ethanol self-administration and of conditioned reinstatement of alcohol-seeking behaviour by the antiopioid peptide nociceptin/orphanin FQ in alcohol-preferring rats. Psychopharmacology (Berl).

[10] Bird MF, Lambert DG (2015). Simultaneous targeting of multiple opioid receptor types. Current opinion in supportive and palliative care.

[11] Azzam AAH, McDonald J, Lambert DG (2019). Hot topics in opioid pharmacology: mixed and biased opioids. British journal of anaesthesia.

[12] Dietis N (2009). Simultaneous targeting of multiple opioid receptors: a strategy to improve side-effect profile. British journal of anaesthesia.

[13] Shen Q, Deng Y, Ciccocioppo R, Cannella N (2017). Cebranopadol, a Mixed Opioid Agonist, Reduces Cocaine Self-administration through Nociceptin Opioid and Mu Opioid Receptors. Frontiers in psychiatry.

[14] de Guglielmo G (2017). Cebranopadol Blocks the Escalation of Cocaine Intake and Conditioned Reinstatement of Cocaine Seeking in Rats. The Journal of pharmacology and experimental therapeutics.

[15] Zheng, F. & Zhan, C.-G. in B*io*lo*gic*s to *T*reat *Substance Use Disorders: Vaccines, Monoclonal Antibodies, and Enzymes* (ed Ivan D. Montoya) 187-225 (Springer (2016).

[16] Zheng, F. *et al*. A highly efficient cocaine-detoxifying enzyme obtained by computational design. *Nature Commun*. **5**, 3457. 3410.1388/ncomms4457 (2014). 10.1038/ncomms4457 PMC3996704 24643289

[17] Smith MA, Greene-Naples JL, Lyle MA, Iordanou JC, Felder JN (2009). The effects of repeated opioid administration on locomotor activity: I. Opposing actions of mu and kappa receptors. The Journal of pharmacology and experimental therapeutics.

[18] Kalivas PW, Duffy P (1993). Time course of extracellular dopamine and behavioral sensitization to cocaine. I. Dopamine axon terminals. *The*. Journal of neuroscience: the official journal of the Society for Neuroscience.

[19] Thomas MJ, Kalivas PW, Shaham Y (2008). Neuroplasticity in the mesolimbic dopamine system and cocaine addiction. British journal of pharmacology.

[20] Beninger RJ (1983). The role of dopamine in locomotor activity and learning. Brain research.

[21] Kalivas PW, Stewart J (1991). Dopamine transmission in the initiation and expression of drug- and stress-induced sensitization of motor activity. Brain research. Brain research reviews.

[22] Sharples SA, Koblinger K, Humphreys JM, Whelan PJ (2014). Dopamine: a parallel pathway for the modulation of spinal locomotor networks. Frontiers in neural circuits.

[23] Mogenson GJ, Jones DL, Yim CY (1980). From motivation to action: functional interface between the limbic system and the motor system. Progress in neurobiology.

[24] Schunk S (2014). Discovery of a Potent Analgesic NOP and Opioid Receptor Agonist: Cebranopadol. ACS medicinal chemistry letters.

[25] Rizzi A (2016). Pharmacological characterization of cebranopadol a novel analgesic acting as mixed nociceptin/orphanin FQ and opioid receptor agonist. Pharmacology research & perspectives.

[26] Schuelke GS, Konkol RJ, Terry LC, Madden JA (1996). Effect of cocaine metabolites on behavior: possible neuroendocrine mechanisms. Brain research bulletin.

[27] Lau CE, Falk JL, King GR (1992). Oral cocaine self-administration: relation of locomotor activity to pharmacokinetics. Pharmacology, biochemistry, and behavior.

[28] Falk JL, Ma F, Lau CE (1991). Chronic oral cocaine self-administration: pharmacokinetics and effects on spontaneous and discriminative motor functions. The Journal of pharmacology and experimental therapeutics.

[29] Tzschentke TM, Rutten K (2018). Mu-opioid peptide (MOP) and nociceptin/orphanin FQ peptide (NOP) receptor activation both contribute to the discriminative stimulus properties of cebranopadol in the rat. Neuropharmacology.

[30] Andersen JM, Ripel A, Boix F, Normann PT, Morland J (2009). Increased locomotor activity induced by heroin in mice: pharmacokinetic demonstration of heroin acting as a prodrug for the mediator 6-monoacetylmorphine *in vivo*. The Journal of pharmacology and experimental therapeutics.

[31] Marquez P, Baliram R, Kieffer BL, Lutfy K (2007). The mu opioid receptor is involved in buprenorphine-induced locomotor stimulation and conditioned place preference. Neuropharmacology.

[32] Yeh SY, Haertzen CA (1991). Cocaine-induced locomotor activity in rats. Pharmacology, biochemistry, and behavior.

[33] Cailhol S, Mormede P (1999). Strain and sex differences in the locomotor response and behavioral sensitization to cocaine in hyperactive rats. Brain research.

[34] Johnson SW, North RA (1992). Opioids excite dopamine neurons by hyperpolarization of local interneurons. The Journal of neuroscience: the official journal of the Society for Neuroscience.

[35] Kuhar MJ, Ritz MC, Boja JW (1991). The dopamine hypothesis of the reinforcing properties of cocaine. Trends in neurosciences.

[36] Wise RA, Bozarth MA (1987). A psychomotor stimulant theory of addiction. Psychological review.

[37] Smith MA (2009). The effects of repeated opioid administration on locomotor activity: II. Unidirectional cross-sensitization to cocaine. The Journal of pharmacology and experimental therapeutics.

[38] Placenza FM, Rajabi H, Stewart J (2008). Effects of chronic buprenorphine treatment on levels of nucleus accumbens glutamate and on the expression of cocaine-induced behavioral sensitization in rats. Psychopharmacology.

[39] Zheng, F. *et al*. Most efficient cocaine hydrolase designed by virtual screening of transition states. *Journal of the American Chemical Society* **130**, 12148-12155, 10.1021/ja803646t (2008). 10.1021/ja803646t PMC2646118 18710224

[40] Zheng, F. & Zhan, C. G. Are pharmacokinetic approaches feasible for treatment of cocaine addiction and overdose? *Future medicinal chemistry* **4**, 125–128, 10.4155/fmc.11.171 (2012). 10.4155/fmc.11.171 PMC4373604 22300091

[41] Zheng F, Zhan C-G (2011). Enzyme therapy approaches for treatment of drug overdose and addiction. Future Med. Chem..

[42] Yao J, Chen X, Zheng F, Zhan C-G (2018). Catalytic reaction mechanism for drug metabolism in human carboxylesterase-1: Cocaine hydrolysis pathway. Mol. Pharmaceutics.

[43] Zhan C-G, Zheng F, Landry DW (2003). Fundamental reaction mechanism for cocaine hydrolysis in human butyrylcholinesterase. J. Am. Chem. Soc..

[44] Pan Y (2005). Computational redesign of human butyrylcholinesterase for anticocaine medication. Proc. Natl Acad. Sci. USA.

[45] Zhan M, Hou S, Zhan C-G, Zheng F (2014). Kinetic characterization of high-activity mutants of human butyrylcholinesterase for the cocaine metabolite norcocaine. Biochem. J..

[46] Hou S, Zhan M, Zheng X, Zhan C-G, Zheng F (2014). Kinetic characterization of human butyrylcholinesterase mutants for hydrolysis of cocaethylene. Biochem. J..

[47] Chen X (2016). Metabolic enzymes of cocaine metabolite benzoylecgonine. ACS Chem. Biol..

[48] Madden JA, Powers RH (1990). Effect of cocaine and cocaine metabolites on cerebral arteries *in vitro*. Life Sci.

[49] Morishima HO, Whittington RA, Iso A, Cooper TB (1999). The comparative toxicity of cocaine and its metabolites in conscious rats. Anesthesiology.

[50] Goldstein RA, DesLauriers C, Burda A, Johnson-Arbor K (2009). Cocaine: history, social implications, and toxicity: a review. Seminars in diagnostic pathology.

[51] Zheng, X., Shang, L., Zhan, C.-G. & Zheng, F. *In vivo* characterization of toxicity of norcocaethylene and norcocaine identified as the most toxic cocaine metabolites in male mice. *Drug Alcohol Depend*. **204**, 107410.101016/j.drugalcdep.102019.107404.107033, 10.1016/j.drugalcdep.2019.04.033 (2019). 10.1016/j.drugalcdep.2019.04.033 PMC7737241 31499241

[52] Chen X (2017). A quantitative LC-MS/MS method for simultaneous determination of cocaine and its metabolites in whole blood. J. Pharm. Biomed. Anal..

[53] Zheng X (2020). Catalytic activities of cocaine hydrolases against the most toxic cocaine metabolite norcocaethylene. Org. Biomol. Chem..

[54] Benuck M, Lajtha A, Reith ME (1987). Pharmacokinetics of systemically administered cocaine and locomotor stimulation in mice. The Journal of pharmacology and experimental therapeutics.

[55] Spiehler VR, Reed D (1985). Brain concentrations of cocaine and benzoylecgonine in fatal cases. Journal of forensic sciences.

[56] Chen X (2016). Long-acting cocaine hydrolase for addiction therapy. Proc. Natl Acad. Sci. USA.

[57] Zheng X (2018). Effectiveness of a cocaine hydrolase for cocaine toxicity treatment in male and female rats. AAPS J..

[58] Zhang T (2017). Clinical potential of an enzyme-based novel therapy for cocaine overdose. Sci. Rep..

